# Arterial Structure and Function in Mild Primary Hyperparathyroidism Is Not Directly Related to Parathyroid Hormone, Calcium, or Vitamin D

**DOI:** 10.1371/journal.pone.0039519

**Published:** 2012-07-16

**Authors:** Margareta Ring, Parastou Farahnak, Tomas Gustavsson, Inga-Lena Nilsson, Maria J. Eriksson, Kenneth Caidahl

**Affiliations:** 1 Department of Molecular Medicine and Surgery, Karolinska Institutet, Stockholm, Sweden; 2 Karolinska University Hospital, Stockholm, Sweden; 3 Department of Clinical Science and Education, Section of Surgery, Karolinska Institutet, Södersjukhuset, Stockholm, Sweden; 4 Chalmers University of Technology, Gothenburg, Sweden; Medical University Innsbruck, Austria

## Abstract

**Objective:**

Elevated levels of calcium and parathyroid hormone (PTH), characteristics of primary hyperparathyroidism (PHPT), may be associated with cardiovascular morbidity and mortality in the general population. We evaluated the possible vascular effects of these risk factors in patients with mild PHPT by using standard methods and new imaging techniques.

**Design:**

A prospective case-control study.

**Subjects and Methods:**

Forty-eight patients with mild PHPT without any known cardiovascular risk factors were studied at baseline and at one year after parathyroidectomy (PTX) in comparison with 48 healthy age- and gender-matched controls. We measured biochemical variables, augmentation index (AIx), aortic pulse wave velocity (PWV_ao_), radial (IMT_rad_) and common carotid artery (IMT_cca_) intima media thicknesses, and the grayscale median (IM-GSM) of the latter.

**Results:**

No significant differences were observed between PHPT patients and controls at baseline for AIx (28.6±12.2 vs. 27.7±12.8%), IMT_rad_ (0.271±0.060 vs. 0.255±0.053 mm), IMT_cca_ (0.688±0.113 vs. 0.680±0.135 mm), or IM-GSM (82.3±17.2 vs. 86.5±15.3), while PWV_ao_ was slightly higher in patients (8.68±1.50 vs. 8.13±1.55, p<0.05). Systolic blood pressure (SBP), calcium, and PTH were higher in patients compared with controls, and decreased after PTX, while vitamin D was lower in patients and increased after PTX. While AIx, PWV_ao,_ IMT_rad_, and IMT_cca_ were related to SBP, neither correlated to vitamin D levels. Only PWV_ao_ correlated weakly to plasma PTH (r = 0.29, p<0.01) and ionized calcium (r = 0.22, p<0.05) but showed no relation when age and SBP were adjusted for.

**Conclusion:**

We found normal arterial function despite high calcium, PTH, and low vitamin D levels, in patients with mild PHPT without cardiovascular risk factors. The cardiovascular risk associated with low vitamin D and/or high PTH and calcium levels may be explained by their coupling to blood pressure and other risk factors rather than direct effects on arterial structure.

## Introduction

Associations between high calcium and parathyroid hormone (PTH) as well as low 25-OH-D levels and cardiovascular risk have been reported in the general population [1], [2], [3], [4]. Recently, elevated PTH levels have been found to predict cardiovascular mortality in patients with coronary disease [5], as well as in the general population [6]. However, other recent data indicate no association between low 25-OH-D levels and all-cause mortality in older men [7].

Primary hyperparathyroidism (PHPT) is characterized by elevated calcium levels, inappropriately increased PTH levels, and is often associated with vitamin D deficiency [8].

Thus, PHPT is a good model to determine the influence of these alterations on the vasculature.

An increased mortality rate has been noted in both unselected PHPT [9], as well as in mild PHPT [10]. However, the majority of previous studies on PHPT include patients with varying disease severity in combination with pre-existing cardiovascular risk factors, which may confound relationships. Nevertheless, it is still unclear whether vascular structure and function are affected in mild PHPT without cardiovascular risk factors [11].

Endothelial function and vasodilatation regulated by nitride oxide (NO) release is of importance in the early atherosclerotic process. Endothelial vasodilatory dysfunction has been described in patients with PHPT [12]. In studies of mild PHPT, the augmentation index (AIx), an indirect measure of the endothelial function and arterial stiffness, has been reported to be increased [13], [14]. Increased pulse wave velocity (PWV_ao_) has been found in PHPT patients with hypertension [15]; however, conflicting results are reported [16]. Carotid intima-media thickness (IMT_cca_) measured by ultrasound is associated with a risk of myocardial infarction and stroke [17], [18]. Alterations in carotid IMT_cca_ have been reported in patients with mild PHPT [19], [20]; however the results are somewhat conflicting [16], [21]. Among PHPT patients with or without cardiovascular risk factors, IMT_cca_ was found to be increased only in the group with risk factors [22]. Recently, echogenicity of the carotid intima media complex has been demonstrated to be related to cardiovascular risk factors [23], and the intima thickness of the radial artery (IMT_rad_) has been shown to be increased in hypertension [24], but none of these indices have yet been studied in PHPT.

With mild PHPT without concomitant disease or known cardiovascular risk factors as a model, our aim was to determine the potential effects of PTH, calcium, and vitamin D on arterial structure and function. Thus, applying the as mentioned novel diagnostic techniques, we compared patients with age- and gender matched healthy controls, and evaluated the vascular effects of normalizing PTH, calcium, and vitamin D by parathyroidectomy (PTX).

## Materials and Methods

### Subjects

In a prospective, previously described [25], [26], case-control study at the Karolinska University Hospital in Stockholm, 410 PHPT patients (319 women) accepted for PTX between January 2006 and November 2008 were consecutively evaluated for possible participation. Patients accepted for PTX were included in the current study if they fulfilled the following criteria: calcium <3.0 mmol/L; no diagnosed hypertension, diabetes mellitus, or renal diseases; no medication affecting the cardiovascular system; no current smoking; BMI <28; and age >18 and <70 years. The majority of our patients had plasma calcium levels below 2.75 mmol/L, and only five patients had plasma calcium levels between 2.76–2.97 mmol/L at the time of inclusion. Fifty-three patients with mild PHPT were consecutively included in the study. Five of the 53 patients were excluded; one man due to later findings of familial hypocalciuric hypercalcaemia, one woman regretted her decision to undergo PTX, one man was excluded because of carotid image storage failure and finally, two men were excluded because of lipid lowering medication. The final population consisted of 48 patients (13 men and 35 women). Of these, 13 PHPT patients had a history of kidney stones (n = 9) and/or osteoporosis (n = 6); while the remaining 35 patients had no classical symptoms coupled to PHPT, 22 of whom were diagnosed with PHPT at a routine health check-up, and the others sought medical attention because of fatigue or diffuse symptoms.

A healthy control group, age- and gender-matched, was randomly selected from the population registry of the city of Stockholm. They were informed by mail and asked to participate in the study. If they fulfilled the inclusion criteria, described above, they were included in the study. If anyone declined, a new randomly chosen control from the population registry was contacted. Two controls were replaced before entering the study: one because of hypertension and the other because of a high PTH level. The median (25, 75 percentiles) time from baseline to follow-up was 1.06 and 1.48 years. One woman did not participate in the vascular examinations at follow-up. The blood pressure and all examinations were performed the morning after an over-night fast, with a resting period of 30–60 minutes in supine position. The same investigator performed the ultrasound examinations and pulse wave analysis. Each participant provided written consent to participate in the study, which was approved by the Local Ethics Committee, Regional Ethical Review Board, EPN, of Stockholm, Sweden.

### Blood Pressure, Body Mass Index

Blood pressure (BP) was measured in both arms using a digital automatic blood pressure monitor Omron M7 (Omron Healthcare Co., LTD, Kyoto, Japan). The mean values of systolic and diastolic blood pressure (SBP and DBP) in both arms were calculated. Mean arterial blood pressure (MAP) was calculated as DBP + (SBP-DPB)/3. Body mass index (BMI) was calculated by dividing weight (kg) by the square of height (m^2^). Body surface area (BSA) was calculated by the formula: BSA (m^2^) = 0.007184 × (weight, kg)^0.425^ × (height, cm)^0.725^.

### Augmentation Index and Pulse Wave Velocity

Augmentation index (AIx) and aortic pulse wave velocity (PWV_ao_) were noninvasively measured using SphygmoCor equipment connected to a computer with SphygmoCor 2000 software (version 7.01, AtCor Medical, Sydney, Australia). Recordings were performed using a single high-fidelity tonometer gently pressed to the radial, carotid, and femoral arteries (SPT-301B, Millar Instruments, Houston, Texas, USA). To measure AIx, the radial artery waves were recorded and processed by the system software. The corresponding aortic pressure waveform was generated from an averaged radial artery waveform using a validated transfer factor [27], [28]. AIx is defined as the difference between the first and the second peaks of the central aortic waveform, expressed as a percentage of the pulse pressure [29], [30]. To obtain PWV_ao_, the pulse waveform was collected sequentially from femoral and carotid artery sites, and by using the R- wave (of a simultaneously recorded electrocardiography, ECG) as a reference, the mean difference in time (ΔT) between sites A (carotid) and B (femoral) was calculated. The surface distance (x_subtracted_) was measured as (carotid-jugulum distance) subtracted from (jugulum-femoral distance). The direct distance over the body surface (x_direct_) was obtained by a standardized formula: x_direct = _0.45x_subtracted_ +0.21* height +0.08 (m) [31]. The use of x_direct_ leads to an overestimation of the real PWV_ao,_ and a scaling factor was used to convert PWV_ao_ distance to “real” PWV_ao._ The real PWV_ao_ was calculated as: 0.8* x_direct_/ΔT, m/s, [32], [33], [34]. The brachial artery blood pressure was measured in connection with the examination, as a mean value of two measurements, by using an automatic monitor Omron M7 (Omron Healthcare Co., LTD., Kyoto, Japan. The radial and aortic blood pressure was calibrated against the brachial artery blood pressure. The aortic AIx and PWV_ao_ are presented as mean values from two recordings.

### Carotid Artery Ultrasound

**Figure 1:**
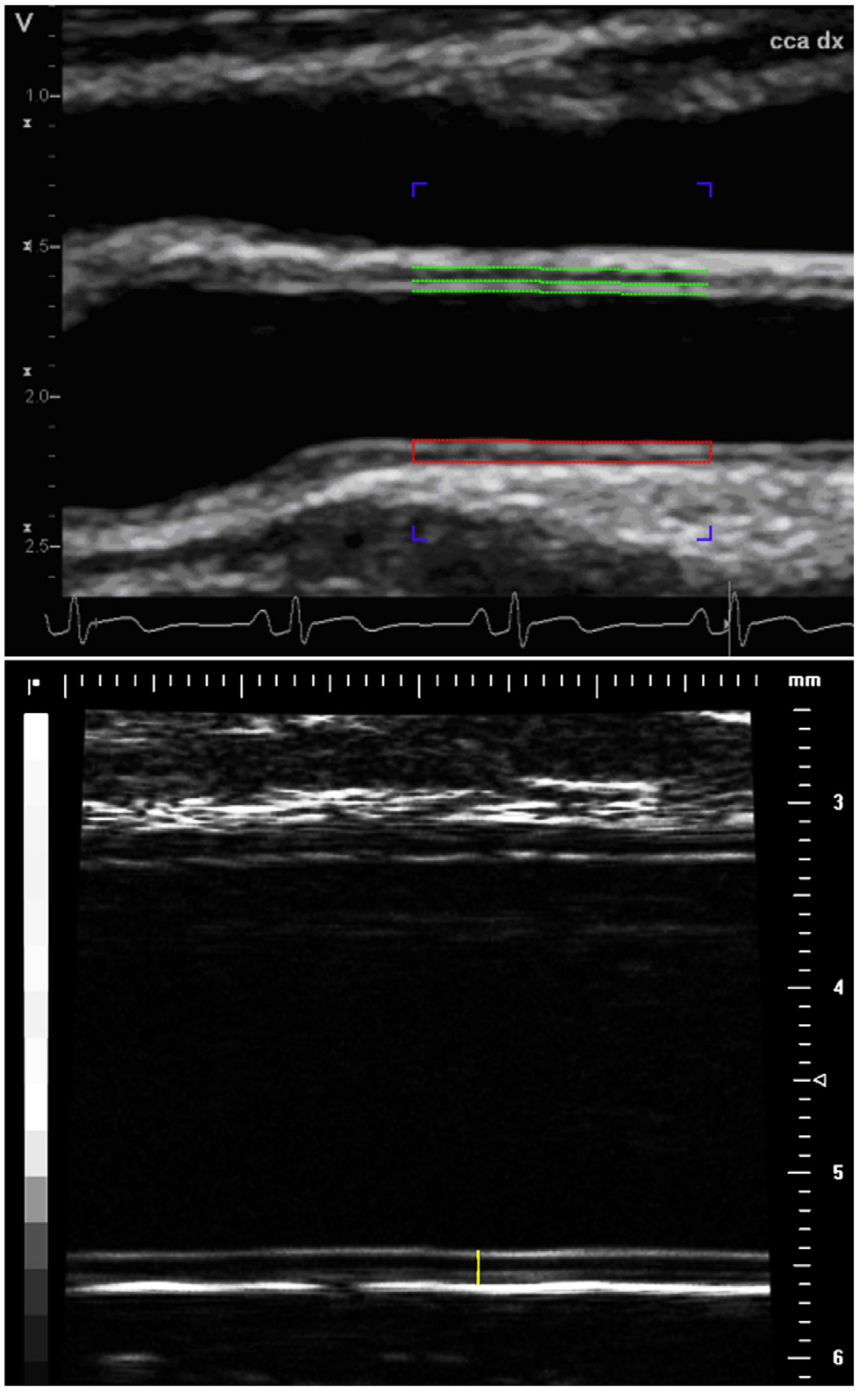
Carotid and radial artery ultrasound images. The red rectangular outline in the upper panel delineates the measurement of the right common carotid artery far wall intima media thickness (IMT_cca_) and grayscale median (IM-GSM). The vertical yellow line in the lower panel indicates the intima media thickness of the radial artery (IMT_rad_).

Two-dimensional images of the common carotid artery (CCA) were acquired using an 8 MHz transducer, 7L, Vivid 7 (General Electric Company, Horten, Norway). The CCA was evaluated 1–2 cm proximal to the carotid bulb. Diastolic images at the time of the electrocardiographic R-wave were stored digitally on EchoPAC (Image Vault 5.0 system, General Electric Company, Horten, Norway). For detection of IMT_cca_ and IM-GSM, six digitized images were imported to validated automated software, Artery Measurement Software (AMS) [35], [36]. AMS was developed in collaboration between the Department of Signals and Systems at Chalmers University of Technology, and the Physiology group at the Wallenberg Laboratory (www.wlab.gu.se), Gothenburg University, Gothenburg, Sweden. A region of interest (ROI) of a 10 mm long segment was manually placed proximal to the carotid bulb, **Figure 1** **, upper panel**. The boarders of the IMT_cca_ of the far wall and the inner lumen diameter (LD_cca_) were identified automatically by the program and the analysis could be manually corrected if necessary. IMT_cca_ was defined as the distance from the leading edge of the lumen-intima interface to the leading edge of the media-adventitia of the far wall. LD_cca_ was defined as the distance from the leading edge of the intima-lumen interface of the near wall and the leading edge of the lumen-intima interface of the far wall [37].

**Figure 2:**
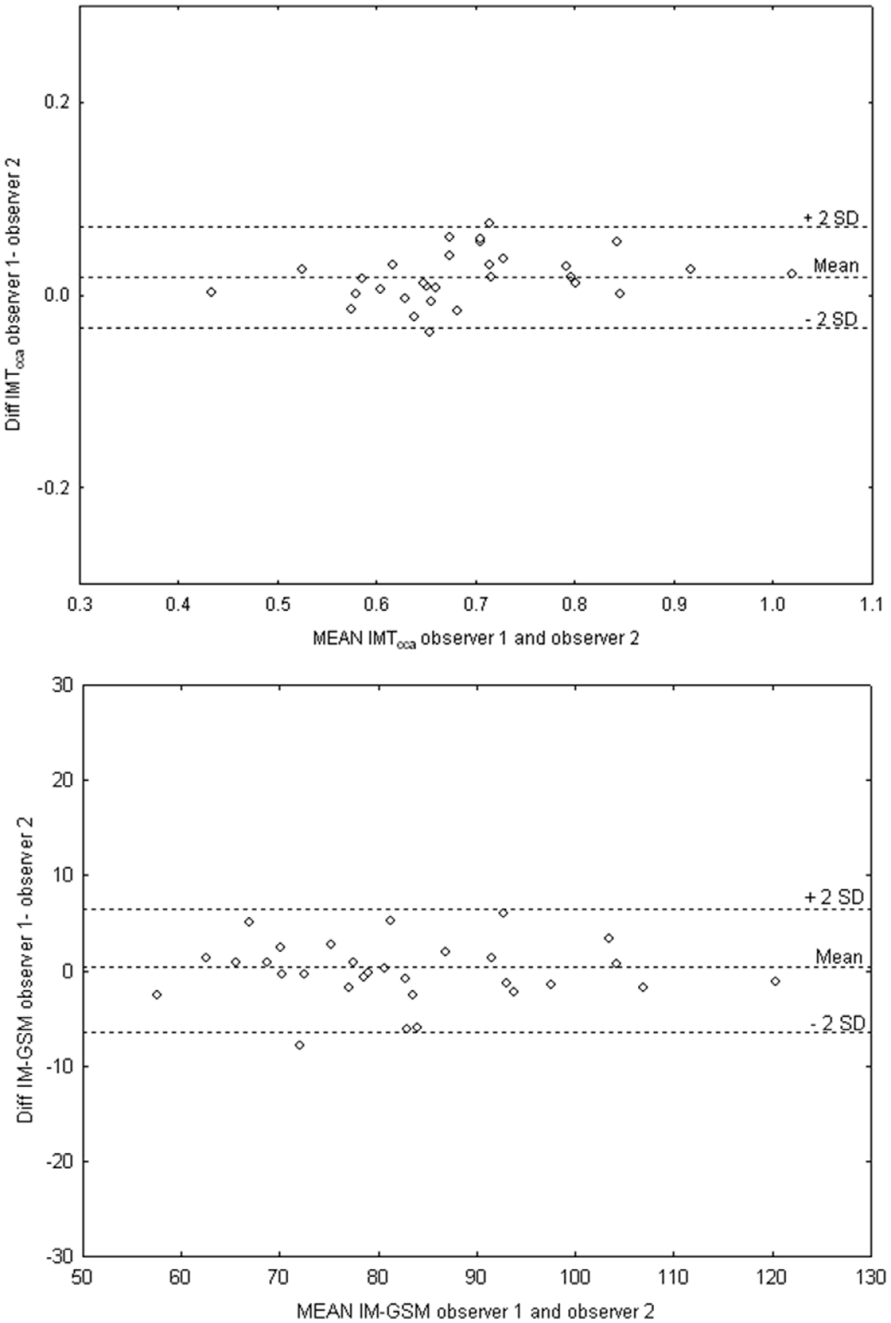
Bland-Altman plots of inter-observer variability. Measurements of the common carotid artery intima media thickness (IMT_cca_) are illustrated in the upper panel, and of the grayscale median (IM-GSM) in the lower panel.

The IM-GSM was calculated in the same intima-media segment of the far wall that was evaluated for IMT_cca_, and from analysis of pixels on a scale from 0 (black) to 255 (white). The adventitia was used as the reference for white and the blood as the reference for black. IMT_cca_, IM-GSM, and LD_cca_ are presented as mean values of six images from both the right and left CCA. Lumen diameter and IMT_cca_ values are shown in mm, while IM-GSM is shown as median gray. At the end of the study, the same investigator performed the image analyses and calculations in random order. Intra- and interobserver variability was measured in 30 randomly selected participants, and the calculations in terms of coefficients of variation (CV) were: 2.90% and 3.80% for IMT_cca,_ 0.40% and 0.70% for LD_cca_, and 2.74% and 3.81% for IM-GSM. Bland-Altman plots for IMT_cca_ and IM-GSM are shown in **Figure 2**.

### High-resolution Ultrasound

A high-resolution ultrasound investigation of the radial artery was performed in 43 patients. The images of the intima media of the radial artery were obtained by high-resolution ultrasound using a 55 MHz transducer (Vevo 770, VisualSonics, Toronto, Canada). B-mode images were recorded from the right radial artery in a longitudinal projection, 1–2 cm proximal to the fold separating the palm of the hand from the forearm. All cine-loops were stored on an external disk and measured offline using VisualSonics software. The thickness of the radial artery intima media (IMT_rad_) and lumen diameter (LD_rad_) were measured according to the leading edge principle [37]. Three sets of measurements were performed from each of the three representative images. The IMT_rad_ of the far wall is presented as a mean value from nine measurements corresponding to the end-diastole, the smallest lumen diameter. The IMT_rad_ was defined as the distance from the lumen-intima interface to the media-adventitia interface, **Figure 1** **, lower panel**. Diastolic LD_rad_ was measured from the M-mode image, defined by the distance between the intima-lumen interface of the near wall and the lumen-intima interface of the far wall. LD_rad_ is presented as a mean value taken from three representative beats. Intra- and interobserver variability in terms of CV were 5.78 and 10.94% for IMT_rad_, and 3.16 and 3.56% for LD_rad_.

### Biochemical Variables

Plasma concentrations of total calcium, creatinine, glucose, high sensitivity C-reactive protein (hsCRP), phosphate, serum concentration of total cholesterol, triglycerides (TG), apolipoprotein A-1 (Apo-A1), and apolipoprotein B (Apo-B) were estimated using Synchron LX® 20 system (Beckman Coulter Inc., Brea, CA, USA). Serum-ionized calcium (Ca^++^) was analyzed on ABL 800 (Radiometer, Copenhagen, Denmark), and plasma concentration of intact parathyroid hormone (PTH) was measured with electro-chemiluminescence immunoassay on the Modular E system (Roche Diagnostics GmbH, Mannheim, Germany). Total serum IGF-1 was analyzed by chemiluminescence immunoassay on DPC Immulite 2000 (Siemens Healthcare Diagnostics, Munich, Germany). Serum concentrations of 25-OH-D were measured by chemiluminiscence on Liason® (DiaSorin, S.p.A, Italy). The plasma concentrations of VWF antigen were determined with latex immunoassay on the BCS XP system (Siemens Healthcare Diagnostics, Munich, Germany). The plasma activity of PAI-1 was measured by a functional enzymatic ELISA assay (Chromolyze PAI-1) from Medinor (Axis-Shield, Dundee, U.K.). The samples of 25 hydroxyvitamin D (25-OH-D), von Willebrand factor (VWF) antigen, and PAI-1 were frozen at −70°C and analyzed in the same series in order to minimize inter-assay variation. Renal function was estimated by calculating the glomerular filtration rate (GFR) according to Cockroft-Gault’s formula: GFR = (140-age in yr) x (weight in kg/creatinine) x (1.23 in men or 1.04 in women).

Vitamin D deficiency was defined as 25-OH-D below 50 nmol/L.

### Statistics

Statistical analyses were performed with PASW Statistics v18 (PASW Inc., Chicago, Illinois, U.S.A.). Data are expressed as mean ± SD. Comparisons between the patients at baseline and the control group were performed with the Mann-Whitney U test for unpaired data, and for intra-individual analyses, Wilcoxon signed rank sum test was used. Spearmańs rank correlation coefficients were computed to assess relationships between variables. Univariate and stepwise multiple linear regression analyses were used to evaluate relationships and adjust for confounders. Results from the multiple regression analyses were presented as standardized beta and adjusted R^2^.

The coefficient of variation (CV), defined as the standard deviation of the absolute differences between the measurements, divided by the mean of two measurements was used to evaluate the intra- and interobserver variability. The reproducibility was illustrated according to Bland-Altman [38]. All tests were two-tailed and p<0.05 was considered to be statistically significant.

## Results

### Clinical Data

Clinical data of healthy controls as well as PHPT patients at baseline and follow-up are presented in **Table 1**. The patients mean age at baseline was 54±8.9 years (range 33 to 68 years). Fifteen patients were below 50 years of age. Forty-five patients had a single parathyroid adenoma, and three had multiglandular disease; median weight of removed tissue 409 mg. Heart rate and diastolic blood pressure did not differ, while systolic blood pressure was slightly higher in patients (p<0.05).

**Table 1:** Clinical data for healthy controls and patients with mild primary hyperparathyroidism before and after parathyroidectomy.

Variable	Controls	PHPT		PHPT Baseline	
		Baseline	Follow-up	vs. Controls	vs. Follow-up
	n = 48 (35 F)	n = 48 (35 F)	n = 48 (35 F)	p-value	p-value
Age, year	54.8±9.0	54.0±8.9	55.3±8.8	0.608	<0.001
HR, bpm	61±9	63±10	60±9^a^	0.324	0.060
SBP, mmHg	119.1±12.6	126.1±15.8	123.3±15.2^a^	0.018	0.010
DBP, mmHg	75.9±7.8	79.8±8.8	78.0±8.2^a^	0.127	0.028
MAP, mmHg	90.3±8.7	95.2±10.2	93.1±9.9^a^	0.025	0.021
Height, cm	169.1±9.3	171.3±9.0	-	0.173	-
Weight, kg	67.5±10.1	70.6±11.4	72.5±12.0^a^	0.155	0.003
BSA, m^2^	1.77±0.18	1.82±0.18	1.84±0.18^a^	0.116	0.004
BMI, kg/m^2^	23.5±2.1	24.0±3.0	24.7±3.3^a^	0.381	0.003

a n = 47. Values are shown as means±SD. F = female, HR = heart rate, bpm = beats per minute, SBP = systolic blood pressure, DBP = diastolic blood pressure, MAP = mean arterial pressure, BSA = body surface area, BMI = body mass index.

### Biochemical Data

Biochemical data are presented in **Table 2**. Calcium and PTH levels were significantly higher and phosphate levels were lower in patients compared with controls and normalized after PTX. The vitamin D levels increased after surgery, but 19 out of 48 patients (40%) had a 25-OH-D level below 50 nmol/l at follow-up, 15±4 months after PTX. There were no significant differences between the subgroups with 25-OH-D levels <50 nmol/L, and >50 nmol/L, regarding SBP, AIx, PWV_ao_, IMT_cca,_ IMT_rad_, IM-GSM, S-Ca, ionized Ca, or PTH levels. Further, these measures did not differ between those with 25-OH-D levels below or above the median (39 nmol/L).

**Table 2:** Biochemical data for healthy controls and patients with mild primary hyperparathyroidism before and after parathyroidectomy.

Variable	Controls	PHPT		PHPT Baseline	
		Baseline	Follow-up	vs. Controls	vs. Follow-up
	n = 48 (35 F)	n = 48 (35 F)	n = 48 (35 F)	p-value	p-value
P-PTH (10–65 ng/L)	44.0±12.5	120.0±40.4	49.6±15.7	<0.001	<0.001
P-Calcium (2.15–2.50 mmol/L)	2.29±0.08	2.61±0.12	2.27±0.08	<0.001	<0.001
S-Ca++ (1.15–1.33 mmol/L)	1.25±0.04	1.46±0.06	1.25±0.05	<0.001	<0.001
P-Phosphate (0.80–1.5 mmol/L)	1.06±0.17	0.85±0.16	1.01±0.17^a^	<0.001	<0.001
Ca*Phosphate	2.43±0.41	2.20±0.39	2.32±0.40	<0.01	0.050
S-25-OH-D (75–250 nmol/L)	64.1±20.6^b^	40.6±17.1^a^	58.2±19.5	<0.001	<0.001
P-Creatinine (<90 µmol/L)	69.1±13.6	66.3±12.1^a^	68.1±15.9^b^	0.434	0.386
GFR, mL/min (>90 mL/min)	93.0±22.0	100.9±22.2^a^	103.1±27.7^b^	0.049	0.861
P-Glucose (4.0–6.0 mmol/L)	5.0±0.34^a^	5.0±0.46^b^	4.9±0.52^a^	0.523	0.471
P-hs-CRP (<3 mg/L)	1.14±1.22^b^	0.92±0.90^a^	1.95±5.05^c^	0.553	0.004
S-Cholesterol (3.9–7.8 mmol/L)	5.71±1.03^b^	5.74±0.80^a^	5.85±0.89	0.857	0.163
S-TG (0.45–2.6 mmol/L)	0.86±0.43^b^	1.00±0.54^a^	0.93±0.51	0.073	0.045
S-Apo-A1 (1.10–2.10 g/L)	1.73±0.32^b^	1.72±0.32^a^	1.69±0.28	0.786	0.760
S-Apo-B (0.50–1.70 g/L)	1.02±0.21	1.01±0.18^a^	1.06±0.17	0.954	0.003
Apo B/Apo-A1 (<1)	0.60±0.14^b^	0.61±0.17^a^	0.64±0.13	0.997	0.117
P-VWF (0.60–1.60 kIE/L)	1.11±0.40^b^	1.05±0.36^b^	1.07±0.40^b^	0.467	0.647
P-PAI-1 (<15 kIE/L)	10.07±6.90^b^	9.51±7.73^b^	8.69±7.49^b^	0.320	0.661
P-Homocysteine (5.0–15.0 µg/L)	9.30±3.00^b^	9.87±2.75^a^	9.59±2.29^a^	0.295	0.266
S-IGF-1 (110–270 µg/L)	137.5±52.9^b^	146.2±48.3^a^	143.6±68.2	0.323	0.086

a n = 47,

b n = 44–46.

c One patient had CRP 35.40 mg/L at follow-up, after excluding the patient, mean hs-CRP was 1.26±1.09 mg/L, p<0.01. Values are shown as means±SD. F = female, P = plasma, S = serum, PTH = parathyroid hormone, S-Ca++ = ionised calcium, S-25-OH-D = 25 hydroxyvitamin D, GFR = glomerular filtration rate. P-hsCRP = high sensitivity C-reactive protein, TG = triglycerides, Apo = apolipoprotein, VWF = von Willebrand factor antigen, PAI-1 = plasminogen activator inhibitor-1 activity, IGF-1 = insulin like growth factor.

### Vascular Data

Vascular structural and functional results are presented in **Table 3**. We found no differences between patients and controls regarding AIx, estimated aortic blood pressure, IMT_cca_, IM-GSM values, or lumen of the CCA and no changes were observed during follow-up. PWV_ao_ was slightly higher in patients 8.68±1.50 (mean±SD) compared to controls 8.13±1.55 (mean±SD), p<0.05, (range 6.6–13.2 for patients and 5.3–13.3 for controls), and did not change during follow-up. The IMT_rad_ did not differ between the groups and remained unchanged at follow-up, while the LD_rad_ was similar to that of controls; however, a marginal increase was noted after one year (p<0.05).

**Table 3:** Augmentation index and ultrasound measurements for healthy controls and patients with mild primary hyperparathyroidism before and after parathyroidectomy.

Variable	Controls	PHPT		PHPT Baseline	
		Baseline	Follow-up	vs. Controls	vs. Follow-up
	n = 48 (35 F)	n = 48 (35 F)	n = 48 (35 F)	p-value	p-value
AIx, %	27.7±12.8	28.6±12.2	29.8±11.9^a^	0.881	0.333
PWV_ao_, m/s	8.13±1.55^b^	8.68±1.50^c^	8.61±1.37^c^	0.027	0.095
Ao SBP, mmHg	109.8±15.3	114.4±17.3	114.8±15.8^a^	0.205	0.473
Ao DBP, mmHg	75.0±9.7	78.1±11.3	78.0±8.7^a^	0.097	0.181
IMT_rad_, mean, mm	0.255±0.053^a^	0.271±0.060^c^	0.271±0.046^b^	0.340	0.623
LD_rad_, mean, mm	1.87±0.35^a^	1.79±0.30^c^	1.90±0.38^b^	0.199	0.020
IMT_cca_, mean, mm	0.680±0.135	0.688±0.113	0.702±0.119^a^	0.354	0.172
LD_cca_, mean, mm	5.94±0.62	6.03±0.45	6.03±0.47^a^	0.229	0.197
IM-GSM, mean	86.5±15.3	82.3±17.2	81.8±15.9^a^	0.091	0.941

a n = 47,

b n = 43–45,

c n = 36–41. Values are shown as means±SD. F = female, AIx = augmentation index, PWV_ao_ = aortic pulse wave velocity, Ao SBP = aortic systolic blood pressure, Ao DBP = aortic diastolic blood pressure, IMT_rad_ = intima media thickness in right radial artery, LD_rad_ = lumen diameter in right radial artery, IMT_cca_ = intima media thickness in both left and right common carotid artery, LD_cca_ = lumen diameter in both left and right common carotid artery, IM-GSM = intima media grayscale median in both left and right common carotid artery.

### Correlations

We evaluated the univariate relations of vascular measures in terms of AIx, PWV_ao,_ IMT_rad_, IMT_cca_, and IM-GSM to baseline data in the complete study cohort of patients and controls in order to verify expected patterns, **Table 4**. As noted, all vascular data except IM-GSM were related to age and systolic blood pressure. Further, correlations of AIx, PWV_ao_, IMT_cca_, IMT_rad_, and IM-GSM with biochemical variables are also presented in *Table 4*. Vascular function was not related to vitamin D. AIx and PWV_ao_ were weakly related to cholesterol, PWV_ao_, and IM-GSM also to PTH, and PWV_ao_ to ionized Ca and triglycerides. None of AIx, PWV_ao_, IMT_rad_, IMT_cca,_ or IM-GSM correlated to P- Phosphate, hsCRP, Apo-B/Apo-A1, Ca*P product, or homocysteine levels.

### Stepwise Regression Analyses

Multiple stepwise regression analyses with indices of vascular function (AIx, PWV_ao_, IMT_cca,_ IMT_rad_ and IM-GSM, one at a time) as dependent variables and demographic data (age, gender, SBP, height, and weight) as independent variables are presented in **Table 5**. More than 30% of the variation in AIx, PWV_ao_, and IMT_cca,_ were explained by age and SBP, in AIx also by gender.

We also analyzed the possible influence of the disease-specific biochemical disturbances (PTH, ionized Ca, phosphate, 25-OH-D) on the five indices of vascular function, one at a time, considering also demographic data, **Table 6**. Apart from a weak relation between AIx and phosphate, no vascular measure was related to the biochemical variables.

## Discussion

In this prospective case-control study of mild PHPT without any known cardiovascular risk factors or medications affecting the cardiovascular system, we found no indication that circulating levels of vitamin D influenced arterial structure or function. Wall composition (IM-GSM) and aortic stiffness (PWV_ao_) were weakly related to PTH and the latter also to ionized calcium levels in univariate analysis, but not when adjusting for confounders like blood pressure. Furthermore, the PTX did not cause any change in indices of vascular function or arterial wall thickness. Our conclusions were substantiated by adding new variables, such as echogenicity of the carotid artery and intima media thickness of the radial artery to the established measures AIx, PWV_ao_, and IMT_cca_.

**Table 4:** Spearman rank order correlations between vascular variables and clinical and biochemical data in the whole group of PHPT patients and controls.

	AIx	PWV_ao_	IMT_cca_	IMT_rad_	IM-GSM
Age, years	0.72***	0.49***	0.51***	0.27*	−0.02
Gender	0.46***	0.04	0.20	−0.02	0.12
SBP, mmHg	0.39***	0.50***	0.44***	0.26*	−0.06
Height, cm	−0.49***	−0.04	−0.19	−0.11	−0.05
Weight, kg	−0.44***	−0.04	0.04	0.07	−0.31**
HR, bpm	−0.13	0.20	0.05	0.06	−0.06
S-Ca++	0.02	0.22*	0.07	0.01	−0.14
P-PTH	0.09	0.29**	0.12	0.13	−0.24*
S-25-OH-D	−0.10	−0.15	−0.05	0.003	0.002
S-Cholesterol	0.27*	0.26*	0.19	0.06	0.12
S-TG	−0.12	0.30**	0.05	0.11	−0.13
P-Phosphate	−0.01	−0.14	−0.003	0.07	0.14
P-Creatinine	−0.31**	−0.22*	−0.16	−0.18	−0.02
PAI-1	0.002	0.24*	−0.07	0.17	−0.16
P-Glucose	−0.09	0.23*	−0.04	0.13	−0.13
GFR	−0.50***	−0.05	−0.16	0.12	−0.28**
IGF-1	−0.46***	−0.09	−0.18	−0.19	−0.13

Statistically significant correlations; * = p<0.05, ** = p<0.01, *** = p<0.001.

AIx = augmentation index, PWV_ao_ = aortic pulse wave velocity, IMT_rad_ = intima media thickness in right radial artery, IMT_cca_ = intima media thickness in both left and right common carotid artery, IM-GSM = intima media grayscale median in both left and right common carotid artery, SBP = systolic blood pressure, bpm = beats per minutes, P = plasma, S = serum, S-Ca++ = ionized calcium, PTH = parathyroid hormone, S-25-OH-D = 25 hydroxyvitamin D, TG = triglycerides, Apo = apolipoprotein.

**Table 5:** Stepwise regression analyses with indices of vascular function as dependent variables, and demographic data as independent variables in the whole group of PHPT patients and controls.

Variable	Model for AIx		Model for PWV_ao_		Model for IMT_cca_		Model for IMT_rad_		Model for IM-GSM	
	R^2^ = 0.57, p<0.001		R^2^ = 0.37, p<0.001		R^2^ = 0.32, p<0.001		R^2^ = 0.08, p<0.01		R^2^ = 0.14, p<0.001	
	β	p-value	β	p-value	β	p-value	β	p-value	β	p-value
Age, years	0.54	<0.001	0.28	<0.01	0.41	<0.001	–	–	–	–
Gender	0.29	<0.001	–	–	–	–	–	–	–	–
SBP, mmHg	0.17	<0.05	0.43	<0.001	0.26	<0.01	0.30	<0.01	–	–
Height, cm	–	–	–	–	–	–	–	–	0.40	<0.01
Weight, kg	–	–	–	–	–	–	–	–	−0.58	<0.001

AIx = augmentation index, PWVao = aortic pulse wave velocity, IMTcca = intima media thickness in both left and right common carotid artery, IMTrad = intima media thickness in right radial artery, IM-GSM = intima media grayscale median in both left and right common carotid artery, SBP = systolic blood pressure.

Thus, although vitamin D deficiency is common in patients with PHPT [8], and is associated with cardiovascular disease in the general population [39], we did not observe any relations between 25-OH-D and AIx, PWV_ao_, IMT_rad_, IMT_cca_, or IM-GSM in our study. Neither were these measures related to other biochemical data specifically abnormal in PHPT, such as Ca^++^ and phosphate, apart from a correlation between PWV_ao_ and PTH and ionized Ca and an inverse correlation between IM-GSM and PTH. Interestingly, Reis *et al*. found no association between vitamin D and carotid IMT apart from a weak association of 25-OH-D levels to internal carotid IMT when adjusting for, but not excluding, hypertension. Further, they found no evidence of an association between 1,25(OH)_2_ D and common carotid IMT or internal carotid IMT, apart from a weak association to internal carotid IMT among patients with hypertension in one of several subgroup analyses [40]. The higher blood pressure among PHPT patients is not clearly understood. Despite the fact that we excluded patients with hypertension, we found significantly higher SBP, although within normal range, in patients compared with controls. SBP decreased after PTX in our patients, while it did not in another series of mild PHPT patients [41]. We have previously reported a weak but significant correlation between SBP and Ca++ as well as PTH levels when including both patients and controls [26]. It has been reported that vitamin D may influence blood pressure through the renin-angiotensin system [42], and a number of possible pathophysiological links to atherosclerosis have been ascribed to vitamin D deficiency, including activation of the renin angiotensin system [43]. Our data add strength to the hypothesis that associations between the atherosclerotic process and circulating levels of vitamin D, ionized Ca, and PTH are mediated through blood pressure and other risk factors. With regard to the relation between vitamin D status and cardiovascular risk, many issues still remain to be resolved. Recent comprehensive reviews do not support a strong link between vitamin D status or supplementation and cardiometabolic outcome or cardiovascular events [44], [45], and another systematic review and meta-analysis, based on 51 trials, found no significant correlation between vitamin D status, hypertension, or cardiovascular mortality [46].

**Table 6:** Stepwise regression analyses with indices of vascular function as dependent variables, and demographic and biochemical data as independent variables in the whole group of PHPT patients and controls.

Variable	Model for AIx		Model for PWV_ao_		Model for IMT_cca_		Model for IMT_rad_		Model for IM-GSM	
	R^2^ = 0.58, p<0.001		R^2^ = 0.37, p<0.001		R^2^ = 0.29, p<0.001		R^2^ = 0.07, p<0.05		R^2^ = 0.18, p<0.001	
	β	p-value	β	p-value	β	p-value	β	p-value	β	p-value
Age, years	0.65	<0.001	0.27	<0.05	0.39	<0.001	–	–	–	–
Gender	0.29	<0.001	–	–	–	–	–	–	–	–
SBP, mmHg	–	–	0.44	<0.001	0.26	<0.05	0.28	<0.05	–	–
Height, cm	–	–	–	–	–	–	–	–	0.37	<0.01
Weight, kg	–	–	–	–	–	–	–	–	−0.63	<0.001
P-PTH	–	–	–	–	–	–	–	–	–	–
S-Ca++	–	–	–	–	–	–	–	–	–	–
P-Phosphate	−0.21	<0.01	–	–	–	–	–	–	–	–
S-25-OH-D	–	–	–	–	–	–	–	–	–	–

AIx = augmentation index, PWV_ao_ = aortic pulse wave velocity, IMT_cca_ = intima media thickness in both left and right common carotid artery, IMT_rad_ = intima media thickness in right radial artery, IM-GSM = intima media grayscale median in both left and right common carotid artery, SBP = systolic blood pressure, PTH = parathyroid hormone, P = plasma, S = serum, S-Ca++ = ionized calcium, PTH = parathyroid hormone, S-25-OH-D = 25 hydroxyvitamin D.

Interestingly, it was recently demonstrated that PHPT causes up- regulation of the matrix metallopeptidase 9 (MMP9) gene and a number of other inflammatory and metabolic genes in fat tissue, with possible impacts on blood pressure and atherosclerosis [47]. In our study of normotensive PHPT patients and controls, we found slightly higher blood pressure and PWV_ao_ in patients. However, data are not unanimous. While *Rosa et al*. noted PHPT patients to have increased arterial stiffness with and without hypertension [48], *Kosch et al*. showed no difference in PWV between PHPT patients and controls, and found no correlations between PWV and increased PTH levels [16].

Our PHPT patients and healthy controls showed no difference in AIx. This is in contrast to the results of Smith *et al.* [13] and Rubin *et al.* [14], who also studied patients with mild PHPT. Smith *et al.* [13] reported higher AIx in patients with mild PHPT compared to controls, and Rubin *et al.* [14] asserted that PHPT was independently associated with increased AIx. In contrast to Rubin *et al.* [14], we did not find any correlation between AIx and elevated PTH. A plausible explanation for the discrepancy is that Rubin *et al.* [14] studied groups containing PHPT patients with hypertension and patients on cardiovascular medication. The association between the severity of PHPT and cardiovascular risk factors, however, is not universal. Similar amount of risk factors was recently reported in hypercalcemic and normocalcemic PHPT patients although cardiovascular and cerebrovascular diseases were more common in hypercalcemic PHPT [49]. In the same study, however, vascular stiffness was not higher in this group than in normocalcemic PHPT or controls, and in congruence with our material unrelated to calcium and PTH.

Probably, cardiovascular diseases combined with PHPT are more likely to affect the arterial wall and IMT than PHPT alone. This is supported by Fallo *et al.* [22], who included PHPT patients with and without cardiovascular risk factors. When compared to healthy controls, only PHPT patients with concomitant cardiovascular risk factors were found to have an increased IMT of the carotid artery wall. Similarly Walker *et al.* [20] reported increased carotid IMT in PHPT patients, including those with cardiovascular diseases, compared to controls. Nuzzo *et al.* [19] excluded subjects with high blood pressure or clinical cardiovascular disease and still found significantly higher carotid IMT values in 20 selected patients compared with controls. However, the authors did not indicate the lipid levels in their study group. Kosch *et al.* [16] found no disturbance in brachial and carotid IMT or aortic PWV in PHPT patients compared to controls, and Lumachi *et al.* [21] reports no improvement in carotid IMT after 18 months of PTX follow-up; further, no correlations between serum calcium and IMT was found. In our study, with careful exclusion of patients with cardiovascular risk factors, we did not observe any differences between patients and controls at baseline, apart from slightly higher SBP and PWV_ao_ in patients with a significant reduction in blood pressure one year after PTX. All our vascular measurements, apart from IM-GSM, were related to age and blood pressure. Adjusting for age and blood pressure, PWV_ao_ was not related to circulating levels of PTH, Ca^++^, phosphate, or 25-OH-D.

In order to investigate the vascular structure more thoroughly, we have used new validated techniques in our study, such as grayscale median for the evaluation of echogenicity of the carotid intima media, and a high- resolution ultrasound for measurement of IMT in the radial artery. We have demonstrated that high- resolution ultrasound allows measurement of very thin structures such as IMT of the rat carotid artery wall [50], and it has also been shown that measurement of the human radial artery IMT is feasible by this technique [24]. In the current study, we measured the intima media thickness in the radial artery and found no significant increase in IMT_rad_ in the PHPT patients compared with controls. By measurement of the grayscale in the intima media images of carotid artery, we tried to obtain information regarding the wall composition, with the assumption that fibrotic infiltration should be more echogenic and lipidous transformation should be more echolucent. Earlier studies have shown that IM-GSM in CCA is closely related to the echogenicity in overt carotid plaques; moreover, the same group further reported a relationship between echolucency in the intima-media complex and cardiovascular risk factors in elderly subjects [23], [51]. However, our study did not indicate abnormal composition of the carotid artery wall as evaluated by this technique.

### Strengths and Limitations

To our knowledge, this is the first study to describe IMT_rad_ and the echogenicity in the intima media of the CCA in patients with mild PHPT. The strict design, excluding cardiovascular risk factors, is the strength of our study, which should favorably influence the reliability of our results. The material is comparatively large for this type of study; however, it is too small for any prognostic evaluations.

We measured the IMT of the common carotid artery in the far wall only, as we assumed relatively symmetrical wall thickness in our patients and controls. Since most patients with PHPT today do not express typical signs and since calcium- and PTH-levels are not examined routinely, it is rarely possible to estimate the duration of the disease.

### Conclusion

We found normal arterial function, despite high PTH and Ca as well as low vitamin D levels, in patients with mild PHPT without cardiovascular risk factors. The cardiovascular risk associated with low vitamin D and/or high PTH and Ca levels may be explained by their coupling to blood pressure and other risk factors rather than direct effects on the arterial structure. Our findings support the importance of adequate blood pressure control in PHPT if PTX is not performed, but do not indicate vascular abnormalities motivating extended follow-up after PTX.
